# Pyloric Obstruction Caused by an Inflammatory Fibroid Polyp

**DOI:** 10.1155/2019/8919204

**Published:** 2019-05-06

**Authors:** Dimitrios Mantas, Nikolaos Garmpis, Damaskini Polychroni, Anna Garmpi, Christos Damaskos, Efstratios Kouskos

**Affiliations:** ^1^Second Department of Propedeutic Surgery, Laiko General Hospital, Medical School, National and Kapodistrian University of Athens, Athens, Greece; ^2^Surgical Department, General Hospital of Mytilini Vostanion, Lesbos, Greece; ^3^Internal Medicine Department, Laiko General Hospital, Medical School, National and Kapodistrian University of Athens, Athens, Greece

## Abstract

This is a case report of a 57-year-old woman who presented with abdominal pain and vomiting over a period of two months. Upper gastrointestinal endoscopies and biopsies were inconclusive, while abdomen computed tomography (CT) scan revealed a large mass arising from the pyloric antrum measuring about 6 × 4.8 cm imitating gastrointestinal stromal tumor (GIST). The patient underwent a laparotomy, and the tumor was totally resected with well-defined borders. The histopathological analysis revealed the mass to be an inflammatory fibroid polyp (IFP).

## 1. Introduction

Inflammatory fibroid polyp (IFP) or Vanek's tumor is a rare benign lesion of the gastrointestinal (GI) tract, first described by Vanek in 1949 as eosinophilic submucosal granuloma [1]. IFPs are formed of connective tissues and vascular structures often with an eosinophilic inflammatory infiltrate, although the histopathology may vary significantly. A number of publications have suggested that trauma, allergic reaction, genetic tendency, radiation, and bacterial, physical, and chemical factors may be inciting factors of the process [2–4]. Therefore, the etiology of IFP is still unknown, but it is accepted that a stimulus, external or internal, results in some individuals developing IFPs.

IFPs usually present in the 5th to 7th decade of life, and children are rarely affected. A familial relationship has also been described [5]. Both genders appear to be equally affected, although there are reports showing a female predominance [6].

IFPs represent less than 1% of all gastric polyps and can be localized anywhere in the gastrointestinal tract but more frequently affect the gastric antrum (66-75%) [7]. They are usually asymptomatic and are found incidentally during endoscopic procedures, laparotomy, or noninvasive imaging methods (e.g., computed tomography (CT)) [3]. When symptomatic, clinical presentation varies in relation to their size and their location. Gastric polyps have been reported to cause abdominal pain, anemia, and vomiting, while patients with IFPs in the small bowel are most likely to present with intussusception and, if left untreated, obstruction with acute abdomen.

This report describes a case of a gastric Vanek polyp, which was surgically completely resected and finally diagnosed with histopathological examination of the resected specimen.

## 2. Case Presentation

A 57-year-old woman presented to the emergency department with acute abdominal pain and nausea. She reported a two-month history of vomiting, postprandial epigastric pain, and weight loss. Past medical history included osteoarthritis, arterial hypertension, and hypothyroidism.

On physical examination, the abdomen was soft and there was no sign of peritonitis. Laboratory studies at admission showed neutrophilic leukocytosis, increased C-reactive protein (CRP), and anemia. Serum electrocytes were within normal limits, while urine examination was clear. No abnormal findings were detected on chest and abdomen radiography. The ultrasound (which was performed as an initial radiologic test in order to check the upper abdomen and confirm or exclude common diseases such as gallstone cholecystitis) revealed a hypoechoic construction with a diameter of 5.6 cm, located at the pyloric antrum.

**Figure 1:**
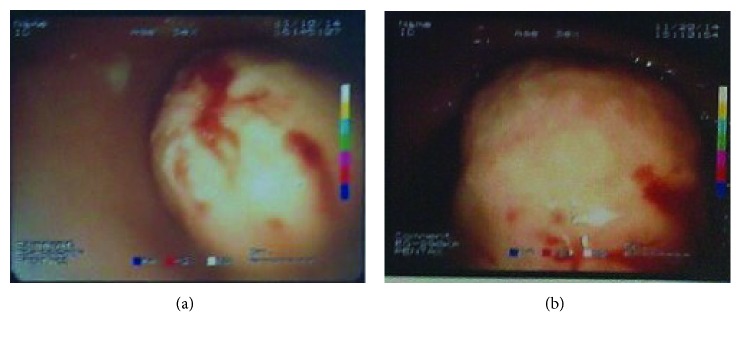
Gastroscopy revealed a round polypoid lesion in the antrum of the stomach measuring about 6 cm.

The patient underwent upper gastrointestinal endoscopy twice, and histopathological examination of biopsy specimens was performed. The lesion appeared to be approximately 6 cm in diameter, obstructing the pyloric antrum and arising from the submucosa or deep mucosa, with well-defined borders (Figures 1(a) and 1(b)). All biopsies were inconclusive most probably due to the submucosal location of the lesion and showed mild-to-moderate inflammation of the gastric mucosa with fibropurulent exudate.

**Figure 2:**
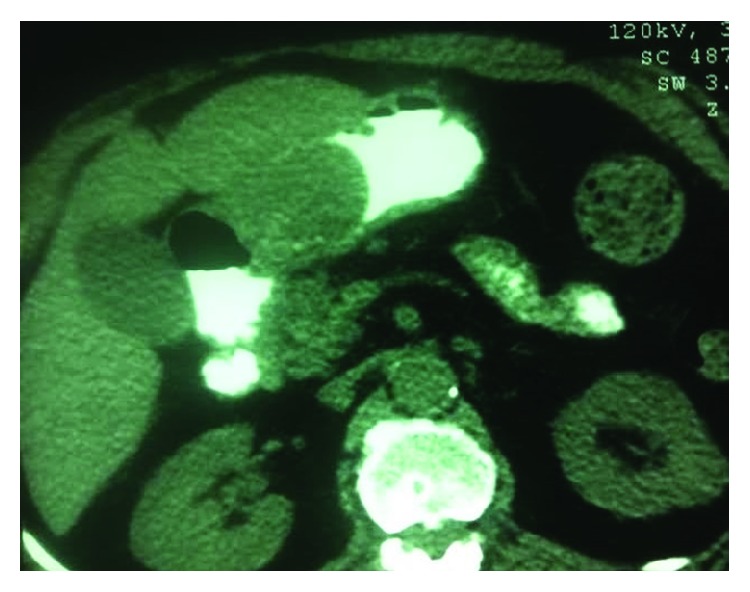
Computed tomography scan of the abdomen demonstrating a well-defined soft tissue mass arising from the antrum.

An abdominal CT scan with administration of oral contrast was performed. It demonstrated a large intraluminal soft tissue mass arising from the pyloric antrum, measuring 6 × 4.8 cm with well-defined borders (Figure 2).

Laparotomy was performed. Through gastrotomy, a propyloric tumor obstructing the antrum was discovered and the lesion was totally excised with macroscopically clear margins. After frozen section biopsy had been performed, the tissue was determined to be suggestive of gastrointestinal stromal tumor (GIST), and the surgery was completed with the occlusion of the gastrotomy. The postoperative course was unremarkable, and the patient was discharged after 8 days in good condition.

Postoperative macroscopic examination of the specimen showed a firm 5 × 4.5 × 4 cm mass partially covered with gastric mucosa. Histopathological analysis showed a myofibroblastic tumor with inflammatory infiltration comprising mainly eosinophils. Among the fibroblasts, blood vessels with concentric arrangement of tumor cells were conspicuous. The benign nature of the mass alongside its submucosal location could not cause easily vessel rupture, explaining the absence of gastrointestinal bleeding despite the size of the finding.

## 3. Discussion

IFPs are rare nonneoplastic lesions arising from the submucosa of the GI tract. Most frequently, they are localized in the gastric antrum. Other less common sites are the other gastric segments, the small bowel, the colon region, the gallbladder, the esophagus, the duodenum, and the appendix [3].

As far as the etiology of IFP is concerned, an abnormal inflammatory response is suspected to be the trigger of this condition, but its mechanical, chemical (such as bile reflux), or biological pathways remain unknown. Some publications suggest that *Helicobacter pylori* infection is a possible etiology [3, 8, 9].

IFPs are often asymptomatic and are diagnosed incidentally on surgical, interventional, or imaging investigations. When symptomatic, the clinical picture is determined by the anatomic location [4]. Patients with lesions in the small bowel are more likely to present with chronic abdominal pain, lower gastrointestinal bleeding, anemia, and more rarely intestinal obstruction due to intussusception. Gastric lesions are usually associated with vomiting, early satiety, upper GI bleeding, and pyloric obstruction. At the time of their diagnosis, they usually measure between 2 and 5 cm, although IFPs up to 20 cm have also been reported [3].

Endoscopically, they appear to be firm, solitary, and well-circumscribed lesions, often ulcerated, deriving from the submucosal layer. Unfortunately, endoscopic biopsies are inconclusive for the diagnosis of IFPs, with only 10% of gastric lesions diagnosed correctly prior to resection [10].

**Figure 3:**
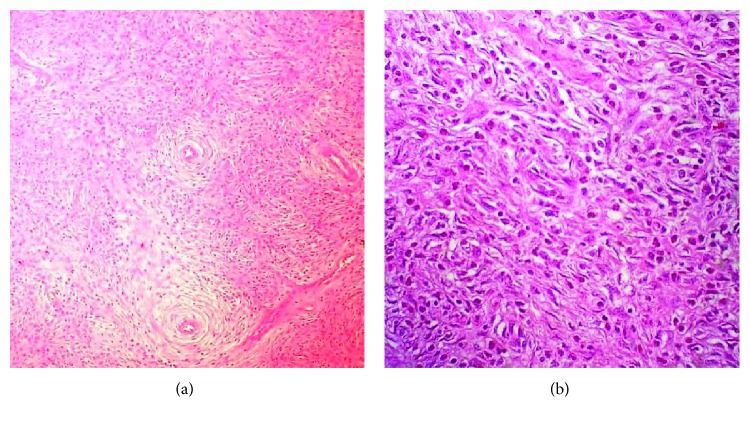
(a) Histological examination of the inflammatory fibroid polyp in the stomach (Η-Ε staining). Middle-power magnification (×100). (b) Histological examination of the inflammatory fibroid polyp in the stomach. High-power magnification (×400). The microscopic view of the polyp showed myofibroblastic cells arranged concentrically around blood vessels, with inflammatory infiltration dominated by eosinophils.

The differential diagnosis includes GIST, submucosal lipoma, and benign mesenchymal tumors such as schwannoma. Sometimes, differentiation is difficult, especially between IFPs and GISTs. Histologically, IFPs are typically characterized by vascular and fibroblastic proliferation with extensive eosinophilic infiltration (Figure 3(a)), but their histopathology may vary. Vessels are usually surrounded by a characteristic concentric arrangement of the fibroblasts with an onion-like appearance [3, 5, 11] (Figure 3(b)). On the other hand, GISTs are intramural tumors that usually lack perivascular concentric cuffing while eosinophilic infiltration is not typical [11, 12].

Immunohistochemistry plays also an important role in tumor classification and can confirm a definite diagnosis. Both tumors are usually positive for CD34 and vimentin, but only GISTs are positive for CD117 [3].

Bearing in mind that IFPs are benign tumors, some authors used submucosal dissection for the treatment of small submucosal tumors, in the case of accessible lesions [8, 13]. Nevertheless, complete resection is still the treatment of choice, because of the size of the lesion and the difficulty to establish a clear diagnosis preoperatively [4, 10]. Following surgical excision, by an endoscopic or open method, Vanek's tumors typically do not recur, making any other adjuvant therapy unnecessary [7].

## 4. Conclusion

In summary, IFPs can cause major pathology, especially chronic episodes of abdominal pain, vomiting, and anemia, mimicking other malignant or benign tumors or other gastrointestinal processes. Patients require emergency hospitalization, and only total excision of the tumor relieves from the symptoms and brings about a definite treatment.
